# Ragweed pollen concentration predicts seasonal rhino-conjunctivitis and asthma severity in patients allergic to ragweed

**DOI:** 10.1038/s41598-022-20069-y

**Published:** 2022-09-23

**Authors:** Maira Bonini, Gianna Serafina Monti, Matteo Maria Pelagatti, Valentina Ceriotti, Elisabetta Elena Re, Barbara Bramè, Paolo Bottero, Anna Tosi, Adriano Vaghi, Alberto Martelli, Giovanni Maria Traina, Loredana Rivolta, Federica Rivolta, Claudio Maria Ortolani

**Affiliations:** 1 Agency for Health Protection of Metropolitan Area of Milan, Milan, Italy; 2 Department of Economics, Management and Statistics, University of Milano-Bicocca, Milan, Italy; 3 Allergy Unit, P.O. Legnano, ASST Ovest Milanese, Milan, Italy; 4 Poliambulatorio Santa Crescenzia, Magenta, Milan, Italy; 5 Istituto Allergologico Lombardo, Casa di Cura Ambrosiana, Cesano Boscone, Milan, Italy; 6 Pneumology Unit, P.O. Garbagnate Milanese, ASST Rhodense, Milan, Italy; 7 Pediatric Unit, P.O. Garbagnate Milanese, ASST Rhodense, Milan, Italy; 8 Department of Pediatrics and Neonatology of Melzo, ASST Melegnano-Martesana, Milan, Italy; 9 Ambulatorio di Allergologia, ASST Lariana, Como, Italy; 10 Fondazione IRCCS Cà Granda Ospedale Maggiore Policlinico di Milano, Milan, Italy

**Keywords:** Environmental sciences, Environmental impact, Respiratory tract diseases, Asthma

## Abstract

In this work, we investigate the correlation between ragweed pollen concentration and conjunctival, nasal, and asthma symptom severity in patients allergic to ragweed pollen using ambient pollen exposure in the Milan area during the 2014 ragweed season We calculate the pollen/symptom thresholds and we assess the effectiveness of ragweed allergen immunotherapy (AIT). A total of 66 participants allergic to ragweed (Amb a 1) were enrolled in the study and divided into two groups: AIT treated (24) and no AIT treated (42). Pollen counts and daily symptom/medication patient diaries were kept. Autoregressive distributed lag models were used to develop predictive models of daily symptoms and evaluate the short-term effects of temporal variations in pollen concentration on the onset of symptoms. We found significant correlations between ragweed pollen load and the intensity of symptoms for all three symptom categories, both in no AIT treated (τ = 0.341, 0.352, and 0.721; and ρ = 0.48, 0.432, and 0.881; p-value < 0.001) and in AIT treated patients ($\tau$= 0.46, 0.610, and 0.66; and ρ = 0.692, 0.805, and 0.824; p-value < 0.001). In both groups, we observed a positive correlation between the number of symptoms reported and drug use. Mean symptom levels were significantly higher in no AIT treated than in AIT treated patients (p-value < 0.001) for all symptom categories. Pollen concentration thresholds for the four symptom severity levels (low, medium–low, medium–high and high) were calculated. Ragweed pollen concentration is predictive of symptom severity in patients with a ragweed (Amb a 1) allergy. Patients treated with AIT had significantly reduced mean symptom levels compared to those without AIT.

## Introduction

**Figure 1:**
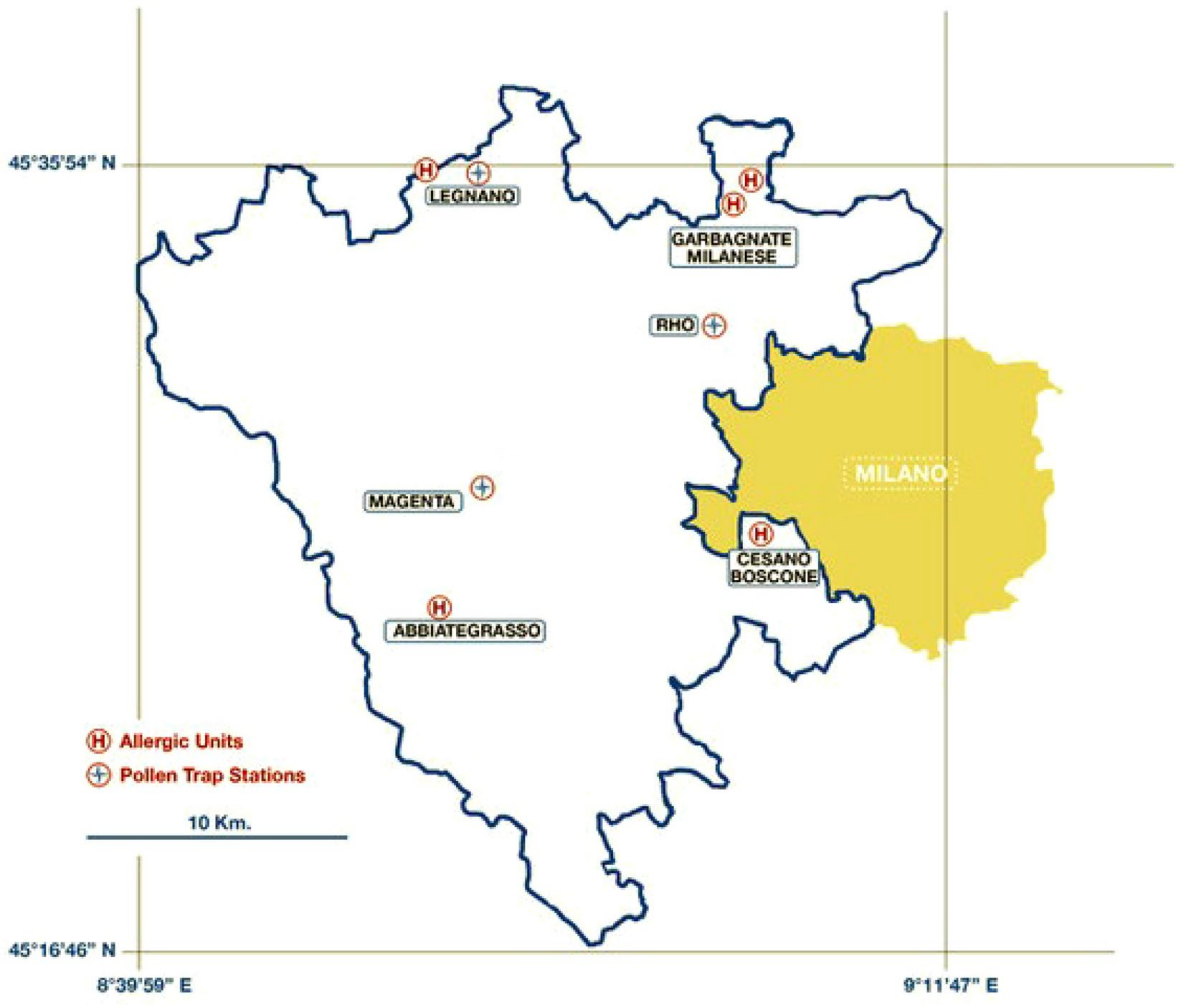
Map of the ASST Ovest Milanese and ASST Rhodense districts, included in the Agency for Health Protection of the Metropolitan Area of Milan (ATS); the ‘blue + ’ symbols represent the three Hirst volumetric traps, and the ‘red H’ symbol shows the location of the five allergy clinics (note: in Legnano there is both an Hirst volumetric trap and an allergy clinic).

*Ambrosia artemisiifolia* (common ragweed) is an invasive plant with highly allergenic pollen that causes seasonal respiratory allergy in people in many countries around the world^1–4^. In particular, the North-West metropolitan area bordering the city of Milan (Italy) is one of the areas in Europe that is most infested with ragweed^5–7^. Currently, the Agenzia di Tutela della Salute (ATS) is the regional agency responsible for healthcare management in the metropolitan area of Milan. The North-West area is comprised of two districts: ASST Ovest Milanese and ASST Rhodense (Fig. 1). Since the 1990s, ragweed plants have spread extensively in this area, becoming the leading cause of rhino-conjunctivitis and asthma in the summer and fall. An epidemiological survey in the municipality of Magenta found that the prevalence of ragweed rhino-conjunctivitis increased in the general population from 9.2 to 14.00% between 1996 and 2005; in the same period, the prevalence of ragweed asthma increased by more than 40%^8^. Furthermore, in 2012, a survey by the ATS Health Agency, involving allergy units in the two districts, found that 54% of patients who visited allergists for the first time for rhino-conjunctivitis and 38% of those hospitalised for asthma were allergic to ragweed^9^. Given the high prevalence of ragweed allergy in this area, it is important to achieve adequate control of rhino-conjunctivitis and asthma symptoms, as well as to limit the social and psychological consequences of the allergy during the ragweed season. Ragweed allergy in this area is a major limiting factor for people in schools and at work, affecting their learning abilities. Consequently, since the 1990s, a series of primary prevention measures have been implemented by the province of Milan and the Lombardy Region in collaboration with the Local Health Agency (today known as the ATS). More recently, these preventive measures have become part of the COST SMARTER action (Sustainable management of *Ambrosia artemisiifolia* in Europe)^10^. In practice, the following measures have been implemented in recent years: (1) control of the territory, for example, through aerobiological monitoring, surveillance, and monitoring of the infested area; (2) urban planning; (3) epidemiological studies; and (4) studies on the effectiveness of various methodologies to limit the spread of ragweed, that is, mowing the weeds before flowering, covering the land, ploughing the soil, harrowing discs, and chemical control^11–15^. Furthermore, since 2013, this area has been infested with a beetle (*Ophraella communa*)^16–21^, which feeds preferentially on ragweed weeds, causing them to dry out and die. As a result of these preventive measures and beetle infestation, in the years following 2013, there was a reduction in both the number of ragweed plants and levels of airborne ragweed pollen^17^.

However, reducing the concentration of pollen in an area does not ensure that there will be a reduction in allergy symptoms in the residents of that area. More specifically, concerning the north-western area of the metropolitan area of Milan, to our knowledge, there are no published studies on the correlation between the concentration of airborne ragweed pollen and severity of allergy symptoms. In other countries, only a few studies have been performed on this topic, and their results are conflicting. Some studies have found a positive correlation between the concentration of ragweed pollen and the severity of both rhino-conjunctivitis and asthma^22–25^, whereas others have not found any correlations^26–28^, and two studies have found an inverse correlation between ragweed pollen concentration and symptom severity^29,30^.

Given the ambiguity of these findings, we planned a cohort study to investigate the correlation between the concentration of ragweed pollen and the severity of seasonal rhino-conjunctivitis and asthma in participants allergic to ragweed. The second aim of our study was to determine the concentrations of ragweed pollen that should be considered as thresholds for the onset and worsening of symptoms. Our third aim was to evaluate the effectiveness of allergen immunotherapy (AIT) in reducing the severity of ragweed allergy symptoms.

## Methods

### Study design

The primary goal of this study was to investigate the association between exposure to airborne ragweed pollen and daily ocular, nasal, and respiratory symptoms, such as asthma, in two subgroups of patients throughout the ragweed season. We monitored individuals sensitised to ragweed and suffering from seasonal ragweed rhino-conjunctivitis with/without asthma from 16 July to 15 September 2014. Study participants were divided into two groups: one consisting of individuals who had never received a ragweed AIT, therefore named “no AIT”, whereas participants in the other group had been treated with ragweed AIT, therefore named “AIT treated”. The patients were treated with AIT prior to and independent of their enrolment in the study. These patients received AIT treatment either in the same year as the study or in the three years immediately preceding the study.

We originally envisioned a different study design. Ragweed and mugwort coexist in the study area, and because mugwort and ragweed blooms partly overlap, we first considered investigating the influence of mugwort exposure on allergy symptoms attributed to ragweed. However, because the number of patients with dual sensitisation (that is, ragweed and mugwort) was small (n = 25), the original study design was modified to include only participants sensitised to ragweed.

From 16 July 2014 to 15 September 2014 all patients included in the study completed a daily clinical diary of symptoms and drugs (CDSD). During the same period, pollen counts were measured from three pollen traps located in the study area (see the ‘Pollen concentration’ section for the exact locations). The daily averages of ragweed and mugwort pollen concentrations obtained from the three pollen traps were used for statistical calculations against the daily mean symptom/drug scores for each of the two subgroups.

### Setting

Patients were recruited and monitored in the areas of the ASST Ovest Milanese and ASST Rhodense districts, both of which are under the Milanese Health Protection Agency ATS (Fig. 1). Both districts are located in the North-West metropolitan area of Milan, covering an area of approximately 827 sq. km (geographical coordinates: to the East: Lat 8° 39′ 59′′ E, to the North: Long 45° 35′ 54′′ E, to the West: Lat 9° 11′ 47′′ E, to the South: Long 45° 16′ 46′′ N (with the exclusion of the municipality of Milan)). Patients within the two districts were enrolled in the allergy units of the following hospitals: Legnano Hospital (Long 45° 35′ 44′′ N, Lat 8° 55′ 23′′ E), Abbiategrasso Hospital (Long 45° 23′ 40′′ N, Lat 8° 54′ 49′′ E), Garbagnate Milanese Hospital, Pneumology and Pediatric allergy units (Long 45° 34′ 53′′ N, Lat 9° 05′ 14′′ E), and Cesano Boscone Ambrosiana Clinic (Long 45° 26′ 57′′ N, Lat 9° 05′ 33′′ E). Each of these five allergy units was located in proximity to one of the three pollen traps (that is, min–max distance: 0.2–19.6 km) (Fig. 1).

### Participants

Participants in the study were a random sample of citizens residing in the designated area with a confirmed diagnosis of ragweed seasonal rhino-conjunctivitis (with or without asthma), some of whom were treated with AIT. A total of 66 participants (32 men and 34 women) were enrolled in the study. The eligibility criteria for participation in the study were as follows: (1) the participants were sensitised to ragweed pollen, demonstrated by a positive skin prick tests (SPT) with commercial ragweed extracts (Lofarma SpA, Milan; ALK-Abellò SpA, Milan) and immunoglobulin (Ig)Es for the total ragweed extract and for rAmb a 1 (pectate lyase) (ImmunoCAP Thermo Fisher Scientific Inc., Monza, Italy). In the case of discordant test results, ragweed sensitisation was confirmed based on positive IgE results for rAmb a 1. (2) The participants had an established and documented history of clinical manifestations of ragweed allergy (that is, symptoms of rhino-conjunctivitis, with or without asthma), coinciding with the ragweed flowering period, in the years immediately preceding enrolment. (3) The participants provided informed consent. (4) Participants were able to adhere to the study protocol and (5) participants remained in their residence throughout the observation period.

All eligible study participants performed SPT with 12 other inhalant allergens, namely pollens of grass, birch, hazel, alder, mugwort, *Dermatophagoides pteronyssinus*, *Dermatophagoides farinae*, *Aspergillus*, *Cladosporium*, *Alternaria*, cat and dog dandruff. Moreover, blood specific IgEs (sIgEs) to mugwort allergen extract, rArt v 1 (recombinant defensin-like protein linked to the polyproline-rich region) and rArt v 3 (recombinant NS LTP type 1) have been determined. Table 1 reports the levels of specific IgEs (mean, ±, sd) to (1) ragweed extract, (2) mugwort extract and to separate recombinant molecular components of ragweed and mugwort pollen. They are (3) rAmb a 1, (4) r Art v 1 and (5) rArt v 3. The averages of specific IgE levels for each allergen extract/component were calculated by gender, age group and subgroup of AIT treated and no AIT treated individuals.

**Table 1:** Specific IgE to ragweed and mugwort extracts, to Amb a 1, Art v 1, and Art v 3 components of 66 participants enrolled in the study.

Patients	N	%	Ragweed extract (kUA/L)			Mugwort extract (kUA/L)			Amb a 1 (kUA/L)			Art v 1 (kUA/L)			Art v 3 (kUA/L)		
			+ /−	Mean	sd	+ /−	Mean	sd	+ /−	Mean	sd	+ /−	Mean	sd	+ /−	Mean	sd
Gender: Total	66	100	66/0	18.88	24.03	56/10	3.43	6.8	66/0	22.2	26.1	22/44	0.78	2.7	8/58	0.18	1.1
Men	32	48.5	32/0	20.63	28.32	26/6	3.0	6.4	32/0	25.1	29.8	10/22	0.55	1.8	4/28	0.07	0.2
Women	34	51.5	34/0	17.24	19.46	30/4	3.7	7.3	34/0	19.4	22.1	12/22	1	3.4	4/30	0.3	3.6
**Age < 19**	18	27.3	18/0	18.8	22.8	15/2	5.6	7.7	18/0	18.2	23.8	6/12	0.98	2.5	4/14	0.1	0.3
19–39	17	25.7	17/0	16.7	24.7	13/4	1.3	1.7	17/0	18.9	25.5	4/13	0.1	0.3	2/15	0.03	0.08
40—59	27	40.9	27/0	18.4	21.2	24/3	2.6	6.1	27/0	21.6	22.9	11/16	1.1	3.7	2/25	0.3	1.7
> 60	4	6.1	4/0	32.1	46.5	3/1	8.1	15.7	4/0	41.0	46.5	1/3	0.7	1.4	0/4	0.007	0.01
No AIT	42	63.6	42/0	14.5	21.2	35/7	3.0	6.3	42/0	16.6	21.7	14/28	0.58	1.7	4/38	0.06	0.2
AIT treated	24	36.4	24/0	26.4	26.6	22/2	4.1	7.6	24/0	29.3	29.0	7/17	1.1	3.9	4/20	0.4	1.8

Most of the participants treated with AIT were sensitised to other pollen, in addition to ragweed, particularly mugwort (that is, 22 positive). Sensitisation to mugwort may have affected the symptoms used to evaluate the effectiveness of AIT for ragweed because the flowering of mugwort and ragweed overlapped in the area during the study period (July–September). We hypothesize, however, that this influence, in the case of our study, was irrelevant because the concentration of mugwort pollen was extremely low in the observation period (Figs. 3 and 4) and, moreover, the average of the IgE values specific for mugwort detected in the patients selected for the study was low (that is, Art v 1 = 1.1 ± 3.9 kUA/L and Art v 3 = 0.4 ± 1.8 kUA/L) (Table 1). Sensitisation to other pollens that provoked pollen allergy in our area (that is, trees and grasses) was irrelevant for the purposes of the study because the load of these pollens during the observation period was absent or irrelevant for the purpose of provoking symptoms. The AIT was not part of the admission/exclusion criteria to allow the enrolled participants to continue the current or already planned AIT. The AIT, both in children/adolescents and adults, was conducted with pre-seasonal or pre-co-seasonal modalities with a duration of 8 weeks, repeating the treatment for three seasons. During the observation period, the AIT participants who had undergone the pre-seasonal procedure had finished treatment, whereas those who had undergone the pre-co-seasonal procedure were in the course of treatment. The mean values of sIgEs to ragweed extract and to Amb a 1 in the subgroups no AIT and AIT were not significantly different. It can be seen in Table 1 that the level of sIgE for ragweed increases in the more advanced age groups. This phenomenon is only apparent, in fact the sample of patients > 60 years consisted of only four individuals of which one had very high values of sIgE to ragweed extract (> 100 kUA/L) and to Amb a 1 (> 100 kUA/L). This discrepancy could be inferred from very high s.d. shown in Table1.

What is more, in accordance with Table 1 patients without AIT treatment always had sIgE levels lower than those who was prescribed with AIT. We did not determine the initial ragweed sIgE values prior to the start of the AIT and therefore we cannot interpret the meaning of the final values. It must also be considered that the overall duration of AIT in our patients was limited, in fact they did not receive a perennial AIT but a pre-seasonal or pre-co-seasonal AIT (limited to 16 weeks/year and then suspended for 36 weeks). Consequently, the known increase in specific IgE in the early stages of current AIT treatment could have influenced the average sIgE levels of the total population^31^. Interestingly, an increase in specific IgE has recently been observed in patients with allergy to pollens (including allergy to ragweed) despite a few years of AIT maintenance with corresponding allergen extracts. The authors interpret this paradoxical increase in sIgE on the basis of environmental changes which can increase the allergenic potency of major allergens in pollens such as ragweed^32^.

To compensate for possible missing data, three periodic examinations were planned. Patients who did not accurately complete the daily diary were excluded from the analysis.

At the end of the recruitment period, from 1 February 2014 to 15 July 2014, 71 participants were assessed using the eligibility criteria. One participant was excluded because he did not undergo the IgE test, and four were excluded because the IgE for rAmb a 1 was negative. The remaining 66 patients were confirmed to be eligible and were included in the study. The mean age was 36.5 years (range: 8–69 years). None of the participants were pregnant or had chronic diseases. All participants resided in the study area, and in the period from 1 February 2014 to 15 July 2014 they had an allergic visit for respiratory symptoms in one of the five allergy units participating in the study. Moreover, 42 participants had never been treated with AIT for ragweed, whereas 24 participants had previously received ragweed AIT. Thus, the patients were divided into two subgroups: no AIT and AIT treated. All recruited participants (100%) completed the follow-up and participated in statistical analysis. The characteristics of the study cohort at enrolment are presented in Table 2.

**Table 2:** Characteristics of the study base at enrolment.

	No AIT n = 42	AIT treated n = 24
Men	23 (55%)	9 (37.5%)
Women	19 (45%)	15 (62.5%)
Mean age at enrolment (range)	34.47 (8–69)	39.58 (11–62)
**Allergy/sensitization**		
rAmb a 1	42/42 (100%)	24/ 24 (100%)
rArt v 1	14/ 42 (33%)	7/24 (29%)
rArt v 3	4/42 (9.5%)	3/24 (12.5%)
Asthma	17/42 (40%)	11/24 (46%)

### Pollen concentration data

Ragweed and mugwort pollen were sampled daily using three Hirst-type pollen traps located in Legnano (45° 35′ 44″ N, Long 8° 55′ 23″ E), Magenta (45° 28′ 16″ N, Long 8° 53′ 33″ E), and Rho (45° 32′ 51″ N, Long 9° 02′ 42″ E), respectively. The Hirst volumetric trap continuously draws 10 L of air per minute onto adhesive-coated tape. Particles in the air stick to the tape, which moves at 2 mm/h to provide a daily sample. The pollen collected from the traps was identified and quantified by a specialised technician and then correlated to the average air volume over 24 h. The reference standard adopted for the sampling and counting of pollen was UNI 11108 of 2004, which was valid at the time of the study.

The following definitions were adopted from the Quality Control Working Group of the European Society of Aerobiology (EAS) and International Association of Aerobiology (IAA)^33^: Pollen count: result of the slide analysis; this quantity is not comparable and must be converted into concentration. Pollen grain: male gametophyte of seed plants. Pollen concentration was expressed as pollen grains/m^3^, that is, the number of pollen grains dispersed in the air per unit of air volume. Main pollen season (MPS): the length of time that pollen is present in the atmosphere at significant concentrations in a place. The MPS in our study was based on the average of the three monitoring stations using the Nillson & Persson criterion^34^: “the period from when the sum of the average daily pollen concentrations reached 5% of the total sum up to the time when the sum reached 95%, that is, the main pollen season with 90% of the entire quantity of pollen collected”. For the purpose of this study, we defined the peak pollen period (PPP) as the period of MPS with higher ragweed pollen concentrations, using 25 pollen/m^3^ as the cut-off value.

### Clinical diary of symptoms and drugs (CDSD)

All study participants were asked to complete the CDSD during the follow-up period. Patients were asked to record their daily symptoms and medication use every evening from 16 July to 15 September 2014. Each symptom was rated on a 4-point scale: 0, no symptoms; 1, mild symptoms; 2, moderate symptoms; and 3, severe symptoms. These ratings were used to score each of the following nine distinct symptoms: nasal, (1) nasal congestion/nasal breathing difficulty and (2) runny nose, (3) itchy nose, (4) itchy throats/ears; ocular, (5) itching and/or burning eyes, (6) tearing and/or wet eyes; bronchial, (7) cough, (8) breathing difficulties while moving and (9) breathing difficulty at rest. Therefore, the total symptom score ranged from 0 to 24, the nasal score ranged from 0 to 9, the ocular score ranged from 0 to 6, and the bronchial score ranged from 0 to 9. Furthermore, patients were also asked to record every single unit of drug taken, that is, the number of tablets/day of oral antihistamines, number of sprays/day of inhaled topical nasal corticosteroids, topical bronchial corticosteroids, topical bronchial corticosteroids, long-acting bronchodilator agents (LABA) or short-acting bronchodilator agents (SABA), and number of drops/day of antihistamine eye drops.

### Patient follow-up during the exposure period

All patients enrolled in the study visited their reference allergy unit immediately before the exposure period began. During the visit, clinical history was collected, a medical examination was performed, and patients provided informed consent for participation in the study. All patients were also provided with a CDSD, which was compiled for all dates during the second visit, after 15 September 2014.

### Comparability of evaluation methods

Patients in both groups, those who were not treated with AIT and those who were AIT treated, were recruited for the study using the same method, underwent the same diagnostic tests, received the same CDSD, were followed up on the same dates, and received the same informative consent.

### Bias

The known overlap in the initial time period of ragweed MPS with mugwort MPS was a possible cause of bias with respect to the symptom score attributed to ragweed, because the symptoms possibly caused by exposure to mugwort pollen were indistinguishable from those caused by ragweed pollen in participants sensitive to both pollen species. However, the concentration of mugwort pollen detected during the study period was very low (see below) and therefore, unlikely to have influenced in any significant way the respiratory symptoms of those patients also allergic to mugwort.

Other possible causes of bias on the symptom/drug score may have been climatic variables, air pollution, any intercurrent respiratory infectious diseases, and the control of symptoms through symptomatic medications (which were not considered to simplify the analyses).

### Ethics

The study protocol complied with the Declaration of Helsinki and all subsequent amendments of Tokyo 1975, Venice 1983, and Hong Kong 1989, as well as with the current regulations for good clinical practice (GCP). The protocol also complied with all national and community regulations applicable to observational studies, and all ethical and deontological principles that inspire the medical profession. This study was approved by the Ethics Committee of Milan Area C (No. 80_122013).

### Quantitative variables

Only ragweed pollen concentration was considered as a feature in the regression models because the correlation between symptom/drug score and mugwort pollen concentration was overall low and not significant.

### Statistical analyses

Spearman's rank ρ correlation coefficient^35^ and Kendall's rank τ correlation coefficient^36^ nonparametric statistical tests were used to examine the correlation between daily ragweed pollen concentrations and symptom intensity, which was measured as the total daily number of symptoms observed in the study participants. We adopted a nonparametric statistical approach because the target variables were not normally distributed.

The nonparametric Kruskal–Wallis test^37^ and the Wilcoxon rank sum test^38^ were used to compare mean symptom level scores between the two groups of patients: the group treated with AIT and the group not treated with AIT.

Time-series analysis was used to analyse the influence of the daily pollen concentration cycle on the onset of allergic symptoms. It is reasonable to assume that the total number of symptoms currently observed depends on the daily pollen concentrations, as well as on the symptom values of the previous day (lag = 1) symptom values. Therefore, we applied a first-order autoregressive distributed lag (ARDL) model to explore these short-run relationships^39^.

${x}_{t}$(t = 1, …,T) represents the daily pollen concentrations and ${y}_{t}$ the total number of symptoms observed in the study patients. The ARDL model is defined as follows:

1 $$y_{t} = \alpha + \phi_{1} y_{t - 1} + \theta_{0} x_{t} + \varepsilon_{t} ,\;\;\left( {{\text{t}} = {1}, \ldots ,{\text{T}}} \right)$$

where ε_t_ is a white noise process, independent of $x_{t}$, $y_{t}$ and $y_{t - 1}$, so that the model (1) can be estimated using ordinary least squares (OLS). The regression coefficients can be interpreted as measures of the influence of the feature on the target variable.

We also considered a first-order autoregressive distributed lag model similar to the model formulated in (1), with the addition of a lagged $x_{t - 1}$ as a further explanatory variable:

2 $$y_{t} = \alpha + \phi_{1} y_{t - 1} + \theta_{0} x_{t} + \theta_{1} x_{t - 1} + \varepsilon_{t} ,\;\;\left( {{\text{t}} = {1}, \ldots ,{\text{T}}} \right)$$

The Akaike information criterion (AIC) and Bayesian information criterion (BIC) were used for model selection. The models with the lowest AIC and BIC values were considered optimal. Finally, because the pollen concentration data showed high variance, we used a square root transformation to stabilise the variance.

All statistical analyses were conducted using R 4.0.2 software^40^.

### Ethics approval

All the procedures performed in this study involving human participants were in accordance with the ethical standards of the national research committee and with the ethical standards as laid down in the 1964 Declaration of Helsinki and its later amendments or comparable ethical standards. The authors confirm that the study was approved by the Bioethics Committee Milano Area C (No 80_122013).

## Results

### Pollen counts

**Figure 2:**
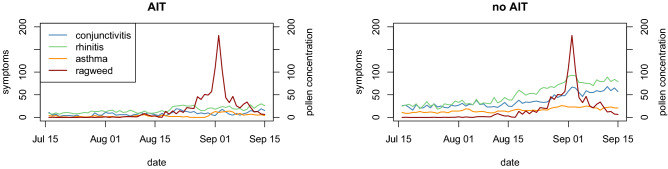
Time series of observed total number of symptoms, for the three categories, in the two groups of patients: AIT treated (left) and no AIT (right), and ragweed mean daily pollen concentrations (secondary Y-axis). Concentrations are provided as daily mean values and are expressed in grains per cubic meter of air (pollen grains/m^3^).

**Figure 3:**
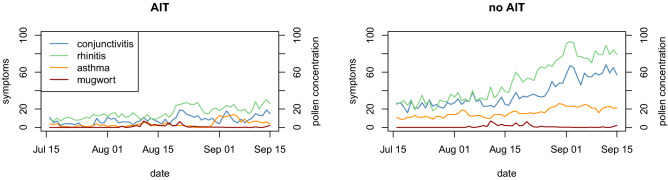
Time series of observed total number of symptoms for the three categories in the two groups of patients: AIT treated (left) and no AIT (right), and mugwort mean daily pollen concentrations (pollen grains/m^3^) (secondary Y-axis).

**Figure 4:**
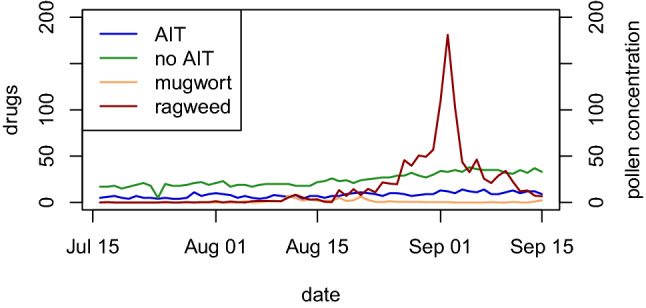
Time series of observed drugs use in the two groups of patients: AIT treated and no AIT, and ragweed and mugwort pollen concentrations (pollen grains/m^3^) (secondary Y-axis).

Figures 2, 3, 4 show the average daily ragweed and mugwort pollen concentrations from the three pollen traps during the follow-up period.

### Ragweed

The ragweed MPS began on 18 August 2014 and ended on 18 September 2014 whereas the peak pollen period (PPP) extended from 25 August 2014 to 10 September 2014 with a cut-off of 25 pollen grains/m^3^. During the MPS, the maximum (C max) and daily mean (C mean) concentrations of ragweed pollen reached by the individual trap stations were as follows: Legnano-trap (C max = 75 pollen grains/m^3^, C mean = 23 pollen grains/m^3^), Magenta-trap (C max = 469 pollen grains/m^3^, C mean = 52 pollen grains/m^3^), and Rho-trap (C max = 145 pollen grains/m^3^, C mean = 27 pollen grains/m^3^). The maximum concentration calculated from the averages of the daily pollen concentrations of the three stations was 211 pollen grains/m^3^ (note that this value does not correspond to the mathematical average of the C max of the individual stations). Because people constantly moved around the studied area, in the analyses, we decided to use the daily average concentration of the three stations.

### Mugwort

The mugwort MPS for 2014 was calculated from the average of the three monitoring stations and ran from 10 August 2014 to 4 October 2014. No significant mugwort PPP was observed because the average C max of the three stations did not exceed the value of 10 pollen grains/m^3^. During the mugwort MPS, we observed a higher concentration of pollen only during two periods: (1) from 11 August 2014 to 21 August 2014 and (2) from 17 September 2014 to 4 October 2014 (which was outside the follow-up time and thus not included in the data). However, these mugwort peaks were low and belonged to two different species (*Artemisia vulgaris* in the first peak and *Artemisia verlotorum* in the second peak).

The mugwort C max recorded by the individual trap stations was as follows: Legnano-trap 10 pollen grains/m^3^, Magenta-trap 16 pollen grains/m^3^, and Rho-trap 15 pollen grains/m^3^. The C max calculated from the averages of the daily pollen concentrations of the three stations was 10 pollen grains/m^3^ (note: this value does not correspond to the mathematical average of the C max of the individual stations).

### Statistical analyses

Table 3 reports the descriptive statistics relating to the average number of daily symptoms per person per symptom category, that is, conjunctivitis, rhinitis, and asthma.

**Table 3:** Descriptive statistics: mean, median, 25% quantile (Q1), 75% quantile (Q3), and standard deviation (sd), related to the average number of daily symptoms per person for each category of symptoms.

Symptoms	Group	Mean	Median	Q1	Q3	sd
Conjunctivitis	No AIT	0.873	0.738	0.595	1.143	0.347
	AIT treated	0.343	0.333	0.208	0.458	0.189
Rhinitis	No AIT	1.201	1.048	0.714	1.768	0.544
	AIT treated	0.673	0.625	0.458	0.865	0.264
Asthma	No AIT	0.385	0.357	0.286	0.500	0.117
	AIT treated	0.157	0.083	0.042	0.208	0.149

Table 4 shows the results of the nonparametric correlation tests for the two groups of patients (no AIT and AIT treated). A moderate significant correlation between ragweed pollen load and symptom intensity was found in the no AIT groups ($\tau =(0.341, 0.352, 0.721)$ and $\rho =\left(0.48, 0.432, 0.881\right),$ p-value < 0.001), whereas a strong significant correlation was found in the AIT treated group ($\tau$ = (0.46, 0.610, 0.66) and $\rho =(0.692, 0.805, 0.824),$ p-value < 0.001) for all three symptom categories. Moreover, in no AIT patients, a moderate to strong positive correlation ($\tau$ = (0.656, 0.711, 0.648) and $\rho$= (0.821, 0.878, 0.818)) with a very high statistical significance (p-value < 0.001) between the number of symptoms and drug consumption was observed; that is, there was strong evidence of a positive monotonic relationship between the two variables in the analysis (see Table 5). In AIT treated patients, this correlation was more moderate but still significant ($\tau$ = (0.495, 0.600, 0.399) and $\rho =($ 0.628, 0.777, 0.516) with a p-value < 0.001).

**Table 4:** Results of the correlation tests between the number of symptoms and pollen concentration: values of Spearman’s ($\rho$) and Kendall’s ($\tau$) rank correlation coefficients and their significance.

Pollen	Symptoms	Group	Spearman		Kendall	
			$\rho$	*p*-value	$\tau$	*p*-value
*ragweed*	Conjunctivitis	No AIT	0.480	< 0.001	0.341	< 0.001
		AIT treated	0.692	< 0.001	0.469	< 0.001
	Rhinitis	No AIT	0.432	< 0.001	0.352	< 0.001
		AIT treated	0.805	< 0.001	0.610	< 0.001
	Asthma	No AIT	$0.881$	< 0.001	$0.721$	< 0.001
		AIT treated	0.824	< 0.001	0.661	< 0.001

	Symptoms		Spearman		Kendall	
			$\rho$	*p*-value	$\tau$	*p*-value
*mugwort*	Conjunctivitis	No AIT	0.293	0.021	0.229	0.018
		AIT treated	0.224	0.080	0.167	0.084
	Rhinitis	No AIT	− 0.003	0.982	− 0.003	0.974
		AIT treated	0.074	0.568	0.035	0.712
	Asthma	No AIT	0.222	0.083	0.132	0.168
		AIT treated	0.083	0.523	0.042	0.668

**Table 5:** Results of the correlation tests the number of symptoms and drug consumption: values of Spearman’s ($\rho$) and Kendall’s ($\tau$) rank correlation coefficients and their significance.

Symptoms	Group	Spearman		Kendall	
		$\rho$	*p*-value	$\tau$	*p*-value
Conjunctivitis	No AIT	0.821	< 0.001	0.656	< 0.001
	AIT treated	0.628	< 0.001	0.495	< 0.001
Rhinitis	No AIT	0.878	< 0.001	0.711	< 0.001
	AIT treated	0.777	< 0.001	0.600	< 0.001
Asthma	No AIT	0.818	< 0.001	0.648	< 0.001
	AIT treated	0.516	< 0.001	0.399	< 0.001

Hereafter, we consider only the ragweed pollen concentration as a feature in the regression models because the correlation between symptom intensity and mugwort pollen concentration was low and overall not significant.

Figures 2 and 3 show the total number of symptoms observed in patients for all three symptom categories and the mean daily ragweed and mugwort pollen concentrations (pollen grains/m^3^) averaged over all three traps. In the no AIT group, the total number of daily symptoms increased over time from mid-July to mid-September, and symptoms increased a few days after the rise in airborne ragweed pollen concentrations, continuing to increase up to their peak value and further due to the known late response to the allergen. We used the Kruskal–Wallis and Wilcoxon rank sum tests to evaluate the hypothesis that mean symptom levels in no AIT patients were greater than those in the AIT treated group. We found significant differences between the daily symptoms of the two groups at a 95% confidence level or higher (Table 6).

**Table 6:** Kruskal–Wallis test and Wilcoxon rank sum test results for comparing no AIT and AIT treated mean symptom levels (alternative = “greater”).

Symptoms	Kruskal–Wallis test			Wilcoxon rank sum test	
	Chi-squared	df	p-value	W	p-value
conjunctivitis	28.397	17	0.041	3840.0	< 0.001
rhinitis	45.513	20	0.001	3737.5	< 0.001
asthma	29.445	12	0.003	3735.5	< 0.001

Figure 4 shows drug use in the no AIT and AIT treated groups, and the daily ragweed and mugwort pollen counts, averaged over the three stations.

Table 7 presents the OLS estimates of the ARLD model (1), considering as covariate the square root of the ragweed pollen daily concentration (pollen grains/m^3^). We also estimated model (2), but selected model (1) based on the AIC and BIC values. We assumed that the target variable ${y}_{t}$, that is, the total number of symptoms at a given day t, may be explained in terms of current ${x}_{t}$, that is, the square root of the ragweed pollen concentration, and past symptom values, ${y}_{t-1}$.

**Table 7:** Estimation of the Autoregressive Distributed Lag (ARDL) model (1) in the two groups of patients for conjunctivitis, rhinitis, and asthma-like symptoms.

Symptoms	Group		Estimate	Std. Error	p-value
Conjunctivitis	No AIT	Intercept	6.098	2.036	0.004**
		${y}_{t-1}$	0.745	0.069	1.49e−19***
		${x}_{t}$	1.221	0.33	0.0005***
		Adjusted R-squared: 0.865; AIC:383.418; BIC: 391.861			
	AIT treated	Intercept	2.444	0.923	0.01*
		${y}_{t-1}$	0.593	0.1040	4.23e−7***
		${x}_{t}$	0.307	0.155	0.052
		Adjusted R-squared: 0.444; AIC:327.397; BIC: 335.841			
Rhinitis	No AIT	Intercept	7.068	2.443	0.005**
		${y}_{t-1}$	0.784	0.069	2.67e−16***
		${x}_{t}$	1.540	0.523	0.005**
		Adjusted R-squared: 0.918; AIC: 407.305; BIC: 415.748			
	AIT treated	intercept	3.219	1.267	0.014*
		${y}_{t-1}$	0.765	0.089	6.07e−12***
		${x}_{t}$	0.269	0.185	0.1517
		Adjusted R-squared: 0.694; AIC:330.904; BIC: 339.347			
Asthma	No AIT	Intercept	3.242	1.06	0.003**
		${y}_{t-1}$	0.743	0.081	8.34e−13***
		${x}_{t}$	0.348	0.132	0.011*
		Adjusted R-squared: 0.844; AIC: 258.518; BIC: 266.961			
	AIT treated	Intercept	0.220	0.341	0.521
		${y}_{t-1}$	0.75	0.076	6.01e−14***
		${x}_{t}$	0.239	0.091	0.011*
		Adjusted R-squared:0.768; AIC:245.455; BIC: 253.899			

Signif. codes: 0 ‘***’ 0.001 ‘**’ 0.01 ‘*’ 0.05 ‘.’ And 0.1 ‘ ’ 1.

**Figure 5:**
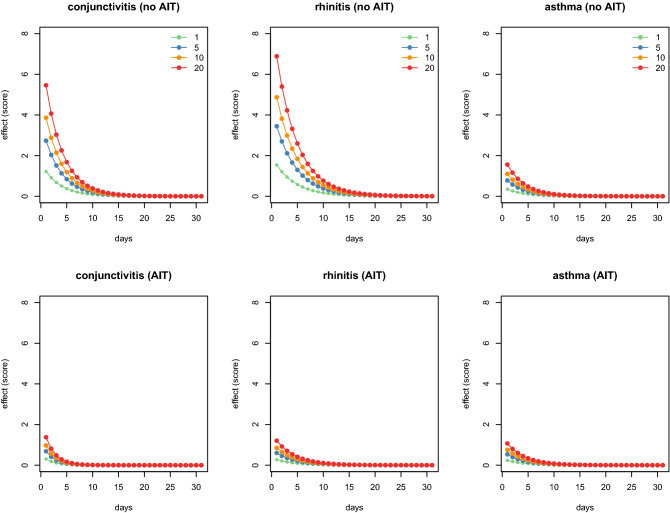
Effects of different increasing levels ℓ (ℓ = 1, 5, 10, and 20) of ragweed pollen concentrations (pollen grains/m^3^) on the total symptoms score in AIT treated and no AIT patients for conjunctivitis, rhinitis, and asthma-like symptoms.

Figure 5 shows the bi-dimensional exposure–response relationship estimated using the ARDL models. Each panel can be read from two different perspectives: it represents the increase in the number of total symptoms in each t + k future day following a single exposure equal to ℓ (ℓ = 1, 5, 10, and 20) ragweed pollen per cubic meter of air at day t = 0 (forward interpretation); otherwise, the contributions of each t − k past day, with ragweed pollen per cubic meter of air equal to ℓ (ℓ = 1, 5, 10, and 20), and the increase in the number of total symptoms at day t (backward interpretation).

To define the airborne ragweed pollen threshold levels for the protection of human health, we considered the quantiles of order (0.25, 0.5, and 0.75) for the total number of symptoms (without distinguishing them in categories) per person in no AIT patients in the period from 1 August 2014 (the first date in which the average number of ragweed pollen was greater than 1) to 2 September 2014 (the ragweed pollen peak).

**Figure 6:**
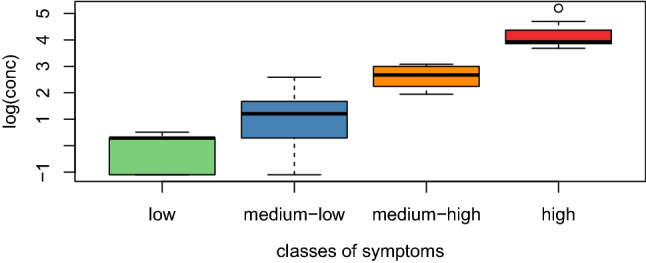
Boxplots of log-transformed ragweed pollen concentrations for the four classes of symptoms, namely low, medium–low, medium–high, and high. The horizontal lines in the boxplots represent the four threshold levels (L) in log-scale.

The quantiles defined four classes of symptoms: low (< 1.81), medium–low [1.81, 2.33], medium–high [2.33, 2.76], and high (> 2.76). The threshold levels (L) of pollen concentrations corresponding to each class of symptoms were obtained by computing the median ragweed pollen concentrations (pollen grains/m^3^) for the corresponding days, that is, L1 = 1.33 pollen grains/m^3^, L2 = 3.33 pollen grains/m^3^, L3 = 14.5 pollen grains/m^3^, and L4 = 50.67 pollen grains/m^3^. Differences in the ragweed pollen concentration distributions (in log-scale) among the four classes of symptoms are depicted as boxplots in Fig. 6 where a clear order among the four distributions is evident.

Data on other sensitisations were not used in the elaboration of the results because they were not significant for the purpose of our study.

## Discussion

### Key results

The first objective of our study was to test the hypothesis that the severity of the symptoms of ragweed seasonal allergy is directly related to the concentration of ragweed pollen in the air. The results of the present study support this hypothesis. First, we found a strong significant correlation in AIT treated patients, and a moderate significant correlation in no AIT patients, between ragweed pollen load and symptom intensity for all three symptom categories (that is, conjunctival, nasal, and bronchial). Moreover, in the no AIT group, we observed a strong positive correlation between the number of symptoms and drug consumption. Notably, correlation does not infer causation, such as whether there are likely causal relationships between the number of symptoms and drug consumption. However, correlational evidence may be useful for prediction. In the AIT treated group, this correlation was more moderate, but still statistically significant. Second, the OSL estimates of the ARLD models (1) demonstrated that daily ragweed pollen concentration is predictive of the severity of each symptom category considered.

The second objective of our study was to establish threshold values of ragweed pollen concentration for different levels of symptom severity. We were able to establish four different ragweed pollen concentration thresholds, each corresponding to a different symptom intensity level: L1 = 1.33 pollen grains/m^3^, L2 = 3.33 pollen grains/m^3^, L3 = 14.5 pollen grains/m^3^, and L4 = 50.67 pollen grains/m^3^.

Our third objective was to test the hypothesis that exposure to equal concentrations of ragweed pollen during ragweed MPS would result in less severe ocular, nasal, and bronchial symptoms in AIT treated patients than in patients not treated with AIT. We found that the mean symptom levels for the three symptom categories were significantly greater over the entire observation period in patients without AIT than in those treated with AIT. The mean daily symptom scores and the mean daily drug consumption scores in the AIT treated group were lower than those in the no AIT group. Furthermore, the ARDL model (1) estimate indicated that the increase in pollen level had a greater contribution to the increase in symptoms in patients without AIT compared to AIT treated.

### Limitations

The main implicit limitation to the first results of the first objective is that the symptom score attributed to ragweed pollen may have been influenced by coexposure to mugwort pollen. This may have occurred both in some participants sensitised to mugwort and in non-mugwort-sensitized participants, due to a cross-reaction between ragweed and mugwort allergens^41^. Of the 66 ragweed-sensitized participants participating in the study, 21 had specific IgE for Art v 1, and 7 for Art v 3. In 2014, mugwort MPS partially overlapped with ragweed MPS during the study’s symptom/drug consumption period (that is, from 16 July 2014 to 15 September 2014). However, during the entire study period, mugwort pollen concentrations were much lower than ragweed concentrations and did not show any peaks. Furthermore, the mean symptom scores were not significantly positively correlated with mugwort pollen concentration values, suggesting that symptom scores did not depend on mugwort pollen concentration. Additionally, the extremely low C max value of mugwort pollen during the 2014 mugwort MPS supports our conclusion that the mugwort pollen concentration did not influence the symptom scores of the study participants.

Other limitations may have arisen from factors that can affect the severity of respiratory symptoms. For example, we did not consider air pollution, which is particularly intense in the study area, because of its high industrial and commercial development. The same applies to possible intercurrent respiratory infections that did not occur to the best of our knowledge, but for which there was no specific provision for reporting them in the CDSD.

The calculated threshold values for ragweed pollen concentration related to symptom severity may also have limitations. It is known that air has allergens contained in pollen grains as well as free allergens in the form of bioaerosols. Studies have found that when the concentration of ragweed pollen is 200 pollen grains/m^3^, contemporarily 2.5 mg/m^3^ of free ragweed allergens are present in the air^42–45^. Consequently, a symptom threshold based on pollen counts alone cannot provide a precise quantification of true exposure to all airborne allergens. Furthermore, atmospheric and environmental factors may have influenced these results. The amount of free allergens associated with pollen grains depends on a number of variables, including air humidity (which can influence the release of allergen-containing starch from pollen grains), and the impact of rain, wind, and pollen destruction in the environment, such as that caused by intense car traffic^45^. Moreover, there is evidence that ragweed pollen collected along high-traffic roads has higher allergenicity than pollen collected in vegetated areas^46^.

Finally, it should be considered that our sample was relatively small; therefore, the reliability of the results could have to be verified in a greater number of individuals. However, this is difficult to realise because it is a complicated process to recruit volunteers.

Concerning the third study objective, possible limits and biases in the results may have occurred because of not considering some of the AIT treatment variables, that is, the definition of the allergenic composition of the extracts used, the maximum and cumulative dose administered, the dose of the main allergens administered, the route of administration, and treatment duration.

Finally, a limitation could have been represented by the different courses of AIT treatment (pre-seasonal and pre-co-seasonal) and the non-homogeneous duration of the pre-co-seasonal AIT (from 1 to 3 years) among the patients in the considered sample with a possible different individual degree of induced tolerance.

### Interpretation

The results of this study indicate that there is a correlation between ragweed pollen concentration and the severity of ocular, nasal, and other respiratory symptom scores in ragweed allergy patients. The data were valid for the experimental conditions of our study, and we excluded a confounding effect due to a coexisting mugwort allergy. Based on the available variables, the correlation data and OLS estimates of the ARLD models (1) showed a strong relationship between ragweed pollen concentration and symptom severity, supporting our main hypothesis.

In a comparative correlation analysis among the three different types of symptoms, we observed a stronger correlation between the number of conjunctivitis and rhinitis symptoms and ragweed pollen concentrations in AIT treated patients than in the no AIT group. This was not the case for the more severe symptoms (that is, asthma), for which we observed a stronger correlation between the number of symptoms and ragweed pollen concentrations in the no AIT group than in the AIT treated patients.

This explanation is also supported by the finding of a strong correlation between the number of symptoms and drug consumption, which was greater in the no AIT than in the AIT treated group. This result may be because conjunctivitis and rhinitis (in patients who have not taken AIT) are treated with OCD, as they are considered less severe symptoms than bronchial ones.

It is likely that AIT treated participants take fewer drugs because they feel protected by immunotherapy. This phenomenon was not observed in patients with bronchial and other respiratory symptoms, in which there was no difference in the correlations between the number of symptoms and pollen concentration in the no AIT and AIT treated participants.

Future studies should consider all the AIT variables (as mentioned above). However, under similar experimental conditions, we did not expect that these additional variables would critically affect the results.

In the literature, most studies investigating the association between pollen load and symptom severity are not specifically aimed at ragweed pollen or have not specifically selected populations of participants allergic to ragweed^26–30^. A few studies that investigated exposure to ragweed pollen in patients with ragweed allergy support our results. Della Valle et al.^24^ found a strong association between daily asthma symptoms, drug scores, and ragweed pollen concentrations in ragweed-sensitized children. They studied a total cohort of 430 children (4–12 years old) sensitised to different pollens, from which ragweed-allergic children had been enucleated and studied separately. Caillaud et al.^25^ also selected only ragweed allergy patients (32 adults) and found a linear relationship between rhinitis, conjunctivitis, asthma symptom scores, and ragweed pollen count. Likewise, Newhouse et al.^22^ found a significant correlation between ragweed pollen, rhinitis, and asthma symptoms in 24 ragweed allergy patients (aged 9–64 years) in Tulsa. The importance of including only patients selectively allergic to the specific pollen under exposure was stressed by Brito et al.^47^, who found a significant correlation between the concentration of olive pollen and the symptoms of rhinitis and asthma in 20 patients mono-sensitized to olive pollen in Ciudad Real.

Other factors that are important to consider when conducting similar studies include the selection of a sufficient number of patients sensitised to ragweed, recruiting only participants with demonstrated sensitisation, placing the pollen traps close to the residences of the participants, and recording both symptom and drug consumption scores.

The results of our study probably depend on the selection of a random sample of patients with both established sensitisation to ragweed and confirmed seasonal respiratory symptoms due to ragweed pollen allergy, residing in a relatively small area (a few km^2^), highly infested with ragweed, and living very close to one of the three pollen traps. Moreover, in the analyses, we used the daily average of the three pollen trap counts, which is likely to be a representative variable of the quantity of pollen that patients were exposed to daily in that area. However, as a pollen exposure of patients at the same time in the environment can be different, we acknowledge that the exact location and the pollen trap height are important factors that could influence the assessment of the thresholds.

Furthermore, even though we recognise its limits, calculating a threshold for symptom pollen levels is an important tool for establishing ragweed-allergy preventative measures. However, comparisons with the results of previous studies have been limited. Although multiple studies have determined symptom thresholds for grass and birch pollen, similar studies on ragweed pollen are practically non-existent. An older study^48^, in which almost all patients with a ragweed allergic rhinitis were symptomatic, proposed a range of 10–50 pollen grains/m^3^ as the threshold level. Another study^49^ established the ragweed pollen concentration threshold for the onset of hay fever symptoms as 1–3 pollen grains/m^3^. Lastly, a study^24^ on environmental triggers of asthma in children found that a threshold of 6–9 weed (not ragweed) pollen grains/m^3^ can trigger asthma symptoms. Unfortunately, the data provided by these studies cannot be compared with ours. Data on ragweed pollen symptom thresholds can be useful for individual prevention only if they are published in conjunction with pollen concentration data. Studies that measure pollen-symptom thresholds along with aerobiological information do exist, but validated criteria on how to provide this information to the public are still lacking^50–53^. However, it would be useful to know the symptomatic thresholds, as they can be used as a standard for all preventative measures carried out in an area, with the aim of reducing ragweed plant expansion.

Finally, the results of our study also support the hypothesis that participants treated with AIT have less severe symptoms upon exposure to the same concentration of ragweed pollen compared to participants not treated with AIT. However, there are limitations to these results owing to some unmeasured AIT variables, such as the characteristics of the ragweed extracts used and the duration and methods of administration. Nonetheless, our general aim was to highlight that AIT can modify the severity of symptoms from exposure to ragweed pollen in treated participants. The model we used can be adopted to evaluate a specific ragweed extract, a particular route of administration, or the duration of AIT. Our experimental model satisfied the requirements cited in the EAACI position paper^54^. Again, comparisons with previously published studies on the “real-world″ effectiveness of AIT are limited. In fact, we were unable to find any published “real-world″ comparative studies of patients treated with AIT pollen versus untreated patients, similar to our study model. We therefore believe that our results are novel. Although not directly comparable to our study, some “real-world″ studies compared patients treated with AIT for different seasonal allergens, for more than one year of treatment, and with untreated control participants. In these studies, AIT effectiveness was confirmed based only on one significant difference in the symptom/drug score between AIT treated participants and controls^55–58^.

### Generalizability

Our data were collected from a population of participants allergic to ragweed (all residing in a well-defined area and highly infested with ragweed plants) and with a high prevalence of seasonal conjunctival, nasal, and bronchial symptoms due to sensitisation to ragweed. Therefore, our results can be generalised to situations with similar contexts. We strongly believe that our finding that ragweed seasonal allergy symptoms are positively correlated with ragweed pollen concentration is generalisable to many areas of the world where there are high concentrations of airborne ragweed pollen. Furthermore, our results have significant implications for the metropolitan area of Milan, as they confirm the validity of the efforts made to control the expansion of ragweed in the territory.

The threshold levels of pollen exposure related to symptom severity established in our study are probably not generalisable. However, these results are consistent with those reported in an earlier study^49^. Given the absolute dearth of confirmatory studies, our data remains unique and can be used as an indicative reference for planning various territorial interventions.

Moreover, because our sample was relatively small, our findings may need to be validated in the same region and with a larger population in the future.

Finally, our findings show that treatment with AIT is able to reduce the symptoms caused by exposure to the allergen; however, it is not able to completely eliminate these symptoms over the short term. We believe that this conclusion confirms the opinions of many experienced allergists regarding AIT.

## Data Availability

The datasets used and analysed during the current study are available from the corresponding author on reasonable request.
